# Chinese school adolescents’ stress experience and coping strategies: a qualitative study

**DOI:** 10.1186/s40359-023-01137-y

**Published:** 2023-03-31

**Authors:** Xiaoyun Zhou, Matthew Bambling, Xuejun Bai, Sisira Edirippulige

**Affiliations:** 1 Centre for Online Health, Princess Alexandra Hospital, The University of Queensland, Building 33, 199 Ipswich Road, Woolloongabba, Brisbane, QLD 4102 Australia; 2 Centre for Health Services Research, The University of Queensland, Brisbane, Australia; 3 Faculty of Medicine, The University of Queensland, Brisbane, Australia; 4 School of Psychology, Australian College of Applied Professions, Brisbane, Australia; 5 Academy of Psychology and Behavior, Tianjin Normal University, Tianjin, China

**Keywords:** Adolescents, Youth, High school, Mental health, Stress, Coping strategies

## Abstract

**Background:**

Stress in adolescence is associated with adverse mental health outcomes. Coping resources have been proved by literature to have buffering effects on the impact of stress on mental health. It is imperative to understand the stress and coping strategies of adolescents. However, to date, there has been a scarce of qualitative examination of stress and coping strategies in adolescents in a Chinese population.

**Objectives:**

This study aimed to understand the stress experience and coping strategies of high school students in China.

**Methods:**

This study adopted a qualitative design involving three focus group interviews. A purposive sampling method was used to recruit high school students who were enrolled in grades 10 to 11, and their teachers, at a Chinese high school which resulted in 20 students and 9 teacher participants. Data were analysed using inductive thematic analysis.

**Results:**

A total of 4 themes were identified: (i) sources of stress; (ii) impacts of stress (iii) coping strategies used by students; and (iv) recommendations for stress management programs. Students experienced excessive stress in their daily lives. The primary source of stress came from high expectations for academic achievement. Other sources of stress were peer relationships and family issues. The stress had negative impacts on students’ emotions, sleep, study, and mental wellbeing. The students demonstrated various coping strategies, with the most common being avoidant coping. Students and teachers agreed that the coping strategies were not effective in reducing stress in the long run and that more coping skills training was needed.

**Conclusions:**

This study is the first to assess the perceptions of Chinese high school students and their teachers regarding adolescent stress experiences and coping strategies. Chinese high school students experienced significant stress in their daily lives and demonstrated unhelpful coping strategies. Participants demonstrated consensus that they did not have the skills to cope. There is a demonstrated need for interventions that focus on increasing coping skills in this population.

**Supplementary Information:**

The online version contains supplementary material available at 10.1186/s40359-023-01137-y.

## Background

Adolescents undergo diverse challenges in their daily lives, including schoolwork, coping with puberty, and building social relationships among peers. These challenges constitute acute or chronic stressors for adolescents. Extensive research has shown that stress has profound impacts on adolescents’ brain development [1], mental health well-being [2, 3]and academic performance [4]. According to the World Health Organization, one in seven adolescents experiences a mental disorder, which accounts for 13% of the global burden of disease in this age group [5]. Previous studies have shown that stress-coping resources have a buffering effect on the impact of stress on mental health [6]. It is therefore imperative to provide adolescents with programs that assist them with developing stress-coping skills. Thus, understanding the stress experience of adolescents and coping behaviours plays a critical role in the development of such programs.

In the past decade, several studies have been conducted in the westernised countries to investigate adolescents’ stress experiences and their coping behaviours. These studies showed that adolescents experienced school life (e.g., long hours of doing homework and materials being too difficult) as the main source of stress [7–12]. Adolescents were also reported to experience stress from sources such as family issues [7, 8, 10, 11, 13], and peer relationships [7, 8, 11, 13, 14]. While there is some research showing students though that some amount of stress could have positive impact on their performance [9, 10] such as increased motivation to do schoolwork and leisure activities and self-esteem [14], most studies have shown that adolescents perceived stress as negative experience which affected their emotional and physical wellbeing [7, 8, 14]. Compared with the amount of literature which investigated the stress experience of adolescents, disproportionately fewer studies have investigated the coping strategies used by adolescents [7–9, 15]. Regardless of the diverse coping strategies reported, students rarely mentioned that they used professional help (i.e., counselling services) as coping strategies. In addition, of particular concern is that the use of maladaptive coping behaviours is common among adolescents, such as internalising stress [9] and engaging with substance use including consumption of alcohol, smoking, marijuana, and other simulants [8, 9].

Previous studies have found that Chinese adolescents experience stress differently from westernised countries, which may be embedded in cultural difference [16]. Chinese adolescents are suggested to show higher mental health issues and lower life satisfaction in comparison with their European counterparts due to the family and education systems’ emphasis on academic performance [17]. According to a national survey, Chinese adolescents are at a high risk of depression, with grades 9 to 11 having at the highest rate of depression. This survey showed that 40% of senior high school students have symptoms of depression, and 11-12.6% of senior high school students have major depressive disorder [18]. However, this is also the age group that are least likely to seek professional help for mental health problems [19]. In addition, previous study also showed that 19.3% of Chinese adolescents had suicidal ideation, and it is significantly associated with stressful life events, among which academic stress and family conflicts were main risk factors [20]. It is therefore essential to understand Chinese adolescent stress experience and coping strategies, so effective programs can be developed and provided. However, at this time no qualitative research has been identified that examines the stress experience and the coping strategies among Chinese adolescents. This study aimed to understand Chinese senior high school students’ experience relating to stress, sources of stress and the impact of stress on their daily life and their coping strategies. As one of the aims of this study was to inform intervention development for Chinese high school students; understanding the needs and preferences of the most important stakeholders—head teachers—is critical for successfully implementing such interventions. In addition, considering it may be difficult for Chinese adolescents to disclose some private stressful experiences, for example, romantic relationships, with stranger peers in focus groups or with the interviewers [21, 22]. Teachers, who usually have years of experience interacting with students, would be a good data source for researchers to comprehensively understand adolescents’ stress experiences [23]. Thus, teachers were involved as an additional data source.

This study will shed light on the Chinese adolescents’ stress and needs and inform program development that aims to manage stress among this population.

## Method

### Study design

This study employed an inductive qualitative design [24] involving focus group interviews. The data were analysed with qualitative thematic analysis. We followed Consolidated Criteria for Reporting Qualitative Research (COREQ) [25] while reporting this study (see Appendix 1).

### Participants

#### Inclusion and exclusion criteria

Participant recruitment occurred in May 2021 at a boarding school in Mianyang city in China. Mianyang city is a moderately sized city located in Sichuan province in southwest China, with a population of around 5.3 million, of which around half live in the urban area. Mianyang city is also an economically moderately developed city with its per capita GDP approximately equal to that of whole China [26]. For students, the inclusion criteria were (i) they should be enrolled in grade 10–11 at the time of interview, (ii) and they should be aged 15–19 years old. After consulting school administrators, as grade 12 students were engaged with preparing for Chinese college entrance examination, involving them will be impractical, therefore, students who were enrolled in grade 12 at the time of interview were excluded. In Chinese high school education system, each class has a head teacher and subject teachers. Unlike subject teachers who are only responsible for teaching a subject (e.g., Mathematics and History), head teachers are responsible for the students’ study and class management, they also interact with the students’ family members constantly. Head teachers have a comprehensive understanding about the study and life of their students and therefore were included as an alternate data source. Subject teachers were excluded.

### Sampling

We used a purposive sampling method to recruit students and teachers. The recruitment.

strategies involved announcement and posters in each classroom by the first author, purpose.

of the study was illustrated. Twenty students and 9 teachers agreed to participate. For the 20.

students, their age ranged from 15 to 17 years old, half of them were female, and half students.

were from grade 10 and the other half were from grade 11. For teachers, the age ranged from 28 to 49 years (mean = 37.9, SD = 6.3). All of them were males, which could be explained by the fact that most head teachers were males in the school. The years of teaching ranged from 5 to 29 years (mean = 15.3, SD = 7.1), slightly more teachers (n = 5) were teaching grade 10 than those who were teaching grade 11 (n = 4) at the time of interview.

### Data collection

Three semi-structured focus group interviews were conducted to collect data. The first focus group involved all 9 teachers. Students (n = 20) were randomly divided into two groups, with each group involved 10 students. The three focus group interviews were conducted by the first author who was a female PhD students with master’s degrees in mental health and psychology, and a male research assistant who was a high school psychology teacher (not within the study site) with master’s degree in psychology at the time of interview. Both of them were trained by experts in qualitative research methods. There were no formal relationships established prior to recruitment of the participants. The focus group interviews were conducted in Mandarin and in person at a meeting room at the school. Three focus group interviews were conducted on three different weekdays at moral education classes where students were normally engaged with non-academic activities.

At the start of each interview, the moderator restated the purpose of the study and asked for permission for recording. Two semi-structured interview guides were developed to understand the experience of teachers and students separately (Table 1). The semi-structured interviews comprised of open questions to facilitate in-depth exploration into adolescents’ stress and coping behaviours. All questions covered the following aspects: (i) what makes adolescents stressed? (ii) how does stress impact adolescents? (iii) what coping strategies do adolescents use to cope with stress? (iv) what are needed from stress management programs? The interview guides were developed by qualitative researchers (MB, SE, XB), who specialised in education, mental health, and psychology. The focus-group interviews lasted between 50 and 65 min. Field notes were made during the interviews. During each focus group interview, before asking the next question, the interviewer summarised the answers from participants to participants, only when there were no more information added, did the interviewer ask the next question. All the interviews were audio-recorded with permission and transcribed and translated to English for subsequent analysis. The transcription and translations were conducted by a professional service, the first author who speaks both Mandarin and English reviewed the transcripts to ensure the quality. Participants were all anonymised by assigning pseudonyms.

**Table 1:** Semi-structured interview guide

Semi-structured Interview Guide
**For teachers**:
1. Do you think students are stressed in their daily life?
2. From your experience with students, what do you think stress students?
3. From your perspective, how does stress impact students?
4. From your experience with students, what do students do to cope with the stress?
5. Do you think students’ coping strategies are helpful in relieving stress?
6. If a professional team can provide a stress management program for high school students, what do you think is needed in this program?
**For students**:
1. Do you feel stressed in your daily life?
2. What do you think is stressing you?
3. How does stress impact you?
4. What do you usually do to cope with the stress?
5. Do you think your coping strategies are helpful in relieving stress?
6. If a professional team can provide a stress management program for high school students, what do you think is needed in this program?

### Data analysis

The qualitative data in this study were analysed using thematic analysis [27]. All the anonymised transcripts were imported to Nvivo 12 software for coding and generating themes. Two authors (XZ & SE) independently performed the analysis. XZ has the background of mental health and school psychology and SE has a background of education and health research. The researchers first familiarise themselves with the data by reading and re-reading the transcripts, notes were made where necessary at this stage. Secondly, initial coding was conducted. To ensure the intercoder reliability, the researchers met regularly during the coding process to double check whether or not they were coding consistently. Adjustments were made as necessary. Third, a coding framework was developed. The initial coding framework was discussed and refined among the research team members. Fourth, themes were generated from the coding framework by grouping codes featuring similar contents, yielding themes that described “source of stress”, “impact of stress”, “coping strategies” and “needs for programs”. Fifth, the analysts reviewed and refined the main themes and identified subthemes. In the final step, the subthemes were refined. The analysis began since data collection. Reflective journals were kept by both analysts throughout the analysis process to reflect how they made decision about which code to use and the process of generating themes. The reflective journal was reviewed by a senior analyst (XB) to ensure that there was no personal bias. XB has a background of psychology and education. The two analysts meet weekly to discuss their codes and themes, any disagreement was resolved by involving a senior qualitative analyst (XB or MB).

## Results

### Results from student interviewees

The results from focus interviews with students were categorised into four themes: [1] sources of stress; [2] impact of stress; [3] stress management; and [4] recommendations for professional help (Table 2). School adolescents’ stress involved various sources including academic stress, conflict with peers and conflict with parents. These sources of stress negatively impacted school adolescents’ emotion, study and sleep. In response to the stress and its impact, students actively used diverse strategies to manage stress, and they thought that these strategies help relieve stress in a short period of time but not in the long run. They, therefore, suggested various recommendations for professional help that will be provided to them.

**Table 2:** Themes and subthemes: student interviewees

**Theme 1: Sources of stress**
Academic stress • Expectations from self, teachers and parents • Difficult to be outstanding in a highly competitive environment
Stress coming from peers • Conflicts with peers • Lack of close friends
Family stress • Parents give too much pressure • Parents are too controlling
**Theme 2: Impacts of stress**
Negative impacts on emotions
Negative impacts on the study
Negative impacts on sleep
**Theme 3: Stress management**
Internalising stress as the primary strategy
Engaging in leisure activities
Talking to friends, teachers, and parents
**Theme 4: Recommendations for stress management programs**
Emotion-regulation and problem-solving skills should be trained
Psychoeducation should be delivered
An understanding and supportive atmosphere should be created

### Theme1: sources of stress

Most adolescents in this study experienced stress. They identified a variety of sources of stress, and all these sources made them feel stressed:

*Then I have a lot of pressure from my grades, from my parents, and from my classmates. I feel that none of them are easy (Zhao, Group one, boy).*

These sources of stress were categorised into three subthemes.

#### Subtheme1.1 academic stress

Most of the stress was experienced at the school. Students felt that there were expectations for them to perform well academically, which included getting high scores in examinations, and ranking top in school. These expectations came from teachers, parents, other family members, and themselves. They wanted to meet these expectations. However, they were not always capable of doing so. When they could not meet others’ expectations, they felt guilty:

*I think my biggest pressure came from my studies. Because, generally, I am aware that our teachers have high expectations for me, I am very afraid of letting them down, and this causes a relatively large psychological pressure… I can’t stand others being disappointed in me. (Qian, Group one, girl)*

*I think my stress comes mainly from the fact that my family’s expectations for me don’t match my actual situation.... my parents have accomplished so much, and they are very successful. I admire them. But they expect me to take another leap of social class as my parents did, but I feel like…according to my current academic performance, it is very unlikely. (Sun, Group two, boy)*

The adolescents experienced academic stress also because high schools are highly competitive environments, they felt that there were too many brilliant students, and it was difficult for them to be outstanding:

*…Sometimes I feel that the difference in intelligence between the genders is too great. It may be true that sometimes hard work can’t make up for this gap. It made me feel very powerless. (Li, Group two, girl).*

It is worth mentioning that, even though, this female adolescent thought that the intelligence difference, especially for science subjects, such as mathematics, physics, and chemistry, may be due to gender differences. Boys in our focus groups also mentioned the intelligence difference caused stress for them.

*I think my stress mainly comes from [examination] grades, studies, and possibly individual intelligence differences. This gap is so big that…. I may have worked hard…. My study time was longer than theirs, and I often felt that when I was studying, they were playing, but in the end, their [examination] grades were better than mine. (Zhou, Group two, boy)*

#### Subtheme1.2 peer stress

Peer-inducted stress is another major concern among our participants. Our participants felt that peer stress came from the fact that they could not find a way to outlet their dissatisfaction or anger amid conflicts with their peers. They felt the need to suppress the feelings in order to maintain good interpersonal relationships at school, which caused stress for them.

*My stress also comes from interpersonal relationships... For example, a while ago, I had an issue with a classmate, …he was in a certain position in our class. He was stopping me from doing something in the classroom, but because the way he managed me was extreme, he frequently punished and yelled at me… I was very angry, although I knew that he was just doing his duty, and I should not blame him, I still felt [angry].. . Because there were times when he criticized me, but not the other person, I felt it was unfair, and that caused stress for me. (Wu, Group one, boy)*

Some of our participants also stated that their interpersonal stress came from the fact that it was difficult to make close friends in high school. They could not talk to anyone when they needed to share their feelings, which caused stress for them.

*In addition to my study, the interpersonal relationship among classmates is also a factor that may cause some pressure on me. Sometimes I felt that even though I had a lot of friends, in some specific situations, it seemed that I had no friends to whom I could turn for help or talk about my feelings. (Zheng, Group two, boy)*

#### Subtheme1.3 family stress

Family is another source of stress identified by our adolescent participants. The school adolescents sometimes had conflicts with their parents, and these conflicts mainly focused on the study, a few conflicts related to peer relationships as well. They felt that their parents tend to put their own pressure on their children. The adolescents felt hurt by the words said by their parents however, they could not refute their parents.

*My grades have been good since I was a child, and my parents had high expectations for my future. Then after entering high school, my test scores were not very ideal, there were too many brilliant students here, and then my parents felt very upset, and then they would vent their dissatisfaction and say how brilliant other people’s children were, and they would not consider how I felt or could I stand that. They would also say some very hurtful words to me, and I felt really hurt, and I would not dare to refute them, because if I really refute them, my parents might cry, etc. (Zhao, Group one, boy)*

*…including aspects like study. Parents always say they are creating a relaxed learning environment for me. What they actually did was putting a lot of pressure on me, making it impossible for me to really get that kind of inner relaxation. (Wong, Group two, girl)*

The school adolescents also felt that their parents were too controlling. They felt that their parents wanted to control every aspect of their life, leaving no privacy for them. This caused a lot of stress on them.

*For example, my parents controlled me very strictly. Then, just like many parents, they say one thing but do opposite things, they said they didn’t control too much about their children’s personal life and kept their children’s private spaces. In fact, it felt like my parents had invaded every aspect of my life, and then I have no privacy at all. (Wong, Group two, girl)*

### Theme 2: impacts of stress

Stress coming from diverse sources together impacted every aspect of school adolescents’ study and life. Even though the interview question asked about the general impact of stress. Almost all students naturally talked about the negative impacts of stress on them. The impacts were categorised into the below subthemes:

#### Subtheme 2.1: negative impacts on emotions

Our student participants felt that they experienced anxiety, upset, low mood, anger, and depression when they felt stressed.

*As the college entrance examination gets closer, there is a feeling that my goal is getting further and further away. Then I got anxious every time I went back to school after holidays. Then I would be very anxious, and sometimes I could not even stop feeling anxious and could not concentrate on studying. (Zhou, Group two, boy) … In fact, it caused a lot of pressure, and many times it still made me feel very anxious. For example, when I was in a class, I felt that I didn’t finish the homework in the last class, and I had to make it up in this class, as a result, I did not finish the homework for this class. At night, when I thought back about the whole day, I felt very upset. (Pang, Group two, boy)*

#### Subtheme 2.2: negative impacts on the study

All our participants agreed that stress had a negative impact on their study. The stress coming from all sources caused a burden for these adolescents. They could not help thinking about the stressful situations, which could be conflicts with parents and peers that happened in the past, which could also be coming examinations. These thoughts impacted their capacity to focus on the homework or lecture they were doing or attending:

*I usually sat there in class and thought about the hurtful things my parents said to me at home, and all those scenes and sounds were repeated in my head, and then I couldn’t concentrate on the study material. (Zhao, Group one, boy)*

The academic stress coming from “intelligence differences” decreased students’ motivation. They felt that regardless of the amount of effort they put into study, it was impossible to catch up with those “intelligent” peers. This gap dampened their self-confidence. As a result, they doubted themselves and did not feel like studying anymore.

*The [intelligence] gap between peers is too big, and it feels like it is something innate. There is no way to make up for it, so I felt that I couldn’t narrow the gap between others through my own efforts…Sometimes I suddenly didn’t want to study anymore. …[silence] I just didn’t want to study anymore. (Qin, Group two, boy) …. Then the gap between me and others is too big, and I might have worked hard, I studied longer than them… and I felt that they were playing while I was studying, but their test results were still better than me…that’s how I began to doubt myself. (Chen, Group two, girl)*

#### Subtheme 2.3 negative impacts on sleep

Two of our student participants felt that stress had caused sleep difficulties for them, which included difficulty in falling asleep, and poor sleep quality.

*…. when I went to school, I sometimes would think of unpleasant things that happened at home, especially when I was resting, for example, it would affect my sleep. (Hu, Group two, girl)*

### Theme 3: stress management

Student participants disclosed a broad range of strategies they used when they felt stressed. Generally, when they felt stressed, they used informal ways to deal with stress and none of the students mentioned the use of professional services, such as mental health services inside and outside school, which were available to them. The stress coping strategies were categorised into three subthemes.

#### Subtheme 3.1: internalising stress as the primary strategy

Almost all student participants mentioned that they tended to suppress the unpleasant feelings when they felt stressed. They might think of the reason why they were stressed, or stay alone turning off their phones, or just had a good cry, as they did not want to bring these unpleasant feelings to others. They also encouraged themselves or blamed themselves while feeling stressed. They thought that these strategies were useful for a short period of time, but the stress will come back:

*[When I’m stressed] I usually went to a place just by myself, and then talked to myself, and turned off the phone because when I was under a lot of pressure, I didn’t like to infect others with my emotions. I usually internalised it by myself. I felt that my regulation of stress was a kind of self-deception, I felt that I had suppressed the feelings, but these feelings might be temporarily suppressed, but they came back after a period of time. (Chen, Group two, girl)*

#### Subtheme 3.2: engaging in leisure activities

Our student participants also indicated that they tried to divert their attention from a stressful situation to leisure activities that they enjoyed. These included a variety of activities, such as doing sports, hanging out and joking with friends, listening to music, having a good sleep, having a bath etc. Some students thought that these strategies help them feel better. However, several students believed that these strategies could help them forget about stress for a short period of time, however, stress remained when fundamental problems were not solved:

*First, I feel that I am an optimistic person and feel less stressed. If I found that I was a little stressed, for example, I was very upset while doing homework, I then washed my face and went to sleep. If it did not work, I had happy friends, then I would spend some time with them doing things irrelevant to studying. I also listened to music in the bed, and then took a shower, which was actually a very good way for me to relieve stress. (Pang, Group two, boy)*

*[With these methods] Most of the pressure can be solved, but some essential problems cannot be solved, such as deep-rooted conflict with parents– ways of thinkings are very different between parents and us. (Meng, Group two, girl)*

#### Subtheme 3.3: talking to friends, parents, and teachers

In addition to relieving stress by themselves and diverting attention to other activities. Our student participants also tended to talk to their friends, as they felt that their friends could listen to them and understand them. They found that talking to friends was an effective way to outlet unpleasant feelings. The same as the above-mentioned strategies, they felt that talking to friends helped them forget about stress for a short period of time, but the stress could come back.

*When I encountered some really stressful situations or felt sad, I would call my best friend to talk about my troubles, and then I would feel much better…But in fact, I felt that I might forget about the stress in the short term, but sometimes if I occasionally see something that resonates, all the previous sadness and pressure would come out again. (Hu, Group two girl)*

Students also turned to their parents and teachers for suggestions and comfort when they felt stressed. If the parents understood them and gave them practical suggestions, the students felt relieved. However, if the parents used the opportunity to judge students, the students would feel worse.

*When I felt stressed about studying and interpersonal relationships, I usually chose to talk to my parents. My parents were the kind of people who understood me. After listening, they would give me a lot of suggestions and comfort me. They would not judge me either, then I wouldn’t feel that stressed anymore. (Wu, Group one, boy)*

*[When I was under pressure] I would also ask my parents or teachers to give me some guidance or advice. Then I felt that [after I told them] they even put more pressure on me, re-stress me, for example, they would say, they believe in me, and then it will be better next time, etc., as a result, I felt more stressed. (Hu, Group two, girl) …Many times when I told my parents about my stress, they would analyse the situation from a different perspective and tried to prove that it was my problem, and it made me very annoyed. (Pang, Group two, boy)*

### Theme 4: recommendations for stress management programs

As this study will inform the development of a stress management program, we asked the student participants what would be needed if we provide a program to them for stress management. Students made a broad range of recommendations that would help them manage their stress. These recommendations were categorised into the following subthemes:

#### Subtheme 4.1: emotion-regulation and problem-solving skills should be trained

Students were aware that they lacked the skills to manage stress. They recommended that we provided skills training for them to regulate their negative emotions when they felt stressed:

*The most important thing is to teach us how to change our moods. In fact, if we thought about stress, it is mostly about emotions. When my parents scolded me, I felt worst at that moment, but after so long, it seemed that there was not so much stress. So, I think the program should teach us how to feel better when we are stressed. (Zhao, Group one, boy)*

They also suggested that problem-solving skills were needed, as they thought it was impossible to reduce stress in the long run if the fundamental problems were not solved.

*I think solving the problem that caused stress for us is the most important thing... The program should teach us how to solve our problems. If the problems are not completely resolved after attending the program, the stress will continue and even get worse. And we may not want to seek help anymore. (Qian, Group one, girl)*

#### Subtheme 4.2: psychoeducation should be delivered

The psychoeducation our student participants needed had four facets. First, our student participants felt that they were not always aware of the fact that they were stressed. Occasionally, they only cried and felt bad. It, therefore, was important to educate them about symptoms of stress so that they knew they were stressed. Second, they felt that they wanted to know the reasons that caused stress for them so that they could figure out ways to reduce their stress. Third, these students felt the need to understand the severity of their stress, in order for them to seek professional help. Fourth, they needed to have knowledge about the resources of mental health services that were available to them.

*For example, one of my friends I knew in our previous dormitory once had some problems, and then she was sad. It was obvious that she needed help. We tried to help her and suggested she talk to the school counsellor. But she kept saying that she was ok. But if we left her to herself, she got more upset… So, I felt that it is important to let us know that we already need to go for help at that stage. And if some students do not want to talk to a school counsellor, where else can we go for such help. (Huang, Group one, girl)*

#### Subtheme 4.3: an understanding and supportive atmosphere should be created

Students agreed that regardless of the type of program that will be delivered, it was of most importance to create an understanding and supportive atmosphere. This atmosphere should comprise of a safe and confidential environment, program-delivers being active listening, non-judgemental, thinking from the students’ perspective, and motivating them to change their status.

*I think it is more important to feel being listened to. I know that some counselling services are face-to-face and one-on-one. Many things are kept confidential, so we feel safe to talk about whatever we want. Sometimes we talked to our teachers and classmates, and they may tell others, and some teachers may tell our parents. (Li, Group two, girl) …When I was stressed, it was because I couldn’t open my heart and mind. And I really wanted to find someone to talk to, I actually hoped that this person could understand and support me no matter I was right or wrong. In fact, many times I knew that I did the wrong thing, but if this person could support me, I would feel much better. (Pang, Group two, boy)*

## Results from teacher interviewees

The results from a focus group interview with teachers were categorised into four themes: (i) the discrepancy between expectations and reality of the source of stress. (ii) impact of stress on students; (iii) coping strategies used by students; and (iv) recommendations for stress management programs (Table 3). Almost all teachers agreed that students were stressed in high school settings. Even though the interviewer and moderator did not give any direction in the discussions, all teachers perceived that the level of stress students experienced had negative impacts on their studies and life. Most teachers thought school adolescents lack the skills to deal with stress. The reasons were: First, parenting methods were inappropriate—either spoiling children or being too strict with children. The children, therefore, did not have a chance to develop the capacity to deal with setbacks. Second, social media had negative impacts—children interacted more with mobile phones than with real people, these adolescents, therefore, did not have adequate experience with interpersonal relationships.

**Table 3:** Themes and subthemes: teacher interviewees

**Theme 1: The discrepancy between expectations and reality is the source of stress**
Academic stress • Expectations from self, teachers and parents • High school is a highly competitive environment • Materials are difficult in high schools
Peer stress • Conflicts with peers • Conflicts with teachers • Suppressing feelings in romantic relationships
Family stress • Conflicts with parents • Lack of love and care
**Theme 2: Impact of stress on students**
Losing motivation in study
Mental health issues
**Theme 3: Coping strategies used by students**
These is no subthemes under theme 3
**Theme 4: Recommendations for stress management programs**
Coping skills training
Career development guidance
Understanding from parents and teachers
Change parents’ perspectives

### Teacher theme 1: sources of stress

Teacher participants thought that there were three main sources that caused stress for the school adolescents. Regardless of the sources that caused stress, overall, teachers thought the stress came from the fact that the expectations did not match the realities. And these sources are illustrated as three subthemes:

#### Teacher subtheme 1.1 academic stress

Teachers all agree that school life was the main reason that caused stress for adolescents. These included examination scores, rankings, and understanding of materials. Teachers all agreed that expectations coming from parents, students and even teachers caused stress for students. Students were expected to get high scores, rank high in examinations, and finally, go to a great university. However, materials in high schools were much more difficult than that in secondary schools. In addition, there were more smart and hardworking students in high schools than in secondary schools. Students who performed well in secondary schools, sometimes including their parents, could not accept the fact that they could not be outstanding anymore. This caused a lot of stress for students.

*The main stress of students comes from study, which is manifested in self-cognition, positioning, and expectations for themselves, especially from the parents of students and the students themselves. They [students and their parents] did not have the right understanding of the students’ intelligence, the studying context, or children’s thinking ability. So, they have high expectations for the students. Then after entering high school, in the stressful competitive learning environment, the results of the students’ efforts may not be as expected, and the conflict between this reality and expectations led to stress. (Fei, teacher group, 42 years old, 20 years of teaching experience)*

#### Teacher subtheme 1.2: peer stress

Teacher participants thought that interpersonal relationships are another main stressor for student adolescents. For student adolescents, their interpersonal relationships included peer relationships, teacher-student relationships, and romantic relationships. The teachers thought that students wanted to maintain good interpersonal relationships, however, they were not adequately equipped with interpersonal skills, especially communication skills, and therefore constantly had challenges in interpersonal life. Students’ interpersonal stress was manifested in three domains. The first domain is conflicts with peers. Adolescents put great importance on trust in peer relationships, they needed peers to keep secrets for them and back up them. When accidental things happened, and they felt betrayed, this caused conflicts with their peers.

*For example, there was a case in our class: in fact, the two students had a very good relationship, but when someone suddenly betrayed him, he was about to explode, and after the eruption, he became severely depressed. (Yue, teacher group, 42 years old, 20 years of teaching experience)*

Another source of interpersonal stress comes from the teacher-student relationship. Teacher participants mentioned that when the students did wrong things, they educated them and sometimes criticized the students. Some students could be very unhappy, but they chose to suppress their feelings instead of communicating with teachers. This caused interpersonal stress for the students.

*There is a student in our class. His teacher criticized him, and then he was depressed for several years. It was not until later in the process of getting along with him that the teacher discovered this problem and took the initiative to talk to him, and he then talked about it, his situation got better after that... The interpersonal stress of students is basically a problem of communication. (Yi, teacher group, 33 years old, 9 years of teaching experience)*

Teachers also mentioned that romantic relationships are another source of interpersonal stress for student adolescents. It is normal for adolescents to have romantic feelings toward others, however, most of the time, they could not express their feelings to that person. One of the reasons was that schools’ rules do not encourage high school students to develop romantic relationships. The other reason was that some students were homosexual, even though the society was not against homosexuals, it did not encourage it either. These students were under more pressure.

*Take a student in our class as an example. He had always liked a girl in our class from secondary school to high school. In addition, the boy was from a divorced family. He needed emotional support very much, but he was a very introverted child. He dared not to tell this girl. Because this girl was a straight-A student and had a very optimistic and cheerful personality. Later, when other students found out, they all laughed at him, saying that he was “unworthy”, and then caused a lot of stress on him. And that girl transited to another school last semester, in the end, he never had the opportunity to express his feeling to that girl. So, he has been very confused and stressed till now. (Wei, teacher group, 38 years old, 17 years of teaching experience)*

Teacher participants thought that the reasons why students lack interpersonal skills were three facets: (i)the excessive use of mobile phones reduced students’ chance to practice interacting with real people; (ii) the interpersonal skills that were demonstrated by television programs and novels were misleading; (iii) many of these students did not have siblings, they lacked natural environment to acquire and practice interpersonal skills at home.

*People like us have siblings, the environment in which we lived and grew up was harsher than today’s adolescents, and we communicated more with real people all the time. Nowadays, children use mobile phones to communicate with each other. When they go home, they play games behind closed doors... (Lin, teacher group, 49 years old, 29 years of teaching experience) … For example, when these children watched some episodes of TV dramas or movies, they felt that “it seems that, in real life, when I deal with interpersonal problems, I can follow the methods in TV dramas and movies”.. . but in real life, they found that the effects they received were diametrically opposite. So, they couldn’t accept it, and they had doubts about this society and the world around them. (Xiao, teacher group, 33 years old, 11 years of teaching experience)*

#### Teacher subtheme 1.3: family stress

Teacher participants thought that family issues are another source of stress for student adolescents. As the school was a boarding school, some students came from places that were far away. The students only went home once per month. They, therefore, lacked in-personal interactions with their parents and lacked love and care that they needed from their parents, which caused stress for them. In addition, teachers thought that the divorce of parents could also cause stress for adolescents.

*… especially in high schools, some students are far away from home. It is this kind of leaving home and parents, lack of care, and sometimes there caused stress for them. (Guo, teacher group, 28 years old, 5 years of teaching experience) … For example, when the relationship among the family members and between the parents are not harmonious, or the parents are divorced. These all kinds of family issues could cause stress for the children, and sometimes… mental diseases. (Sheng, teacher group, 37 years old, 14 years of teaching experience)*

### Teacher theme 2: impacts of stress on students

Teachers thought that the amount of stress the high school students were experiencing mainly had two kinds of impacts. The first impact was related to study, and the second impact was related to students’ mental health.

#### Teacher subtheme 2.1: losing motivation in study

Teacher participants thought that students had unrealistic expectations for their study outcomes, and they lacked the ability to deal with setbacks. They could lose motivation to study when stressed.

*Many students thought that they were genius because they did very well from elementary school to secondary school. However, after entering high school, they found that there were many problems in the study that he could not solve. At that time, they began to doubt themselves, lost motivation, and even gave up on themselves. (Fei, teacher group, 42 years old, 20 years of teaching experience)*

#### Teacher subtheme 2.2: mental health issues

Teacher participants agreed that stress could cause severe mental health issues, such as depression, self-harm, suicide attempts etc. for the students.

*A girl in our previous class, had physical issues, such as cervical spondylitis, and lumbar spondylitis, but that girl liked to study very much, but after sitting in the classroom for a long time, she was always in pain. She wanted to continue with her study… receiving acupuncture and moxibustion, but all of these made her very painful. This caused her to want to commit suicide, to end all of this. Later, in the third year of high school, there was more stress from studying, and her thought of committing suicide became stronger. (Yuan, teacher group, 43 years old, 20 years of teaching experience) … Later, he felt that his mother didn’t trust him, so he adopted a very extreme method, that was, self-harm, cutting his wrists. (Zhang, teacher group, 38 years old, 13 years of teaching experience)*

### Teacher theme 3: coping strategies used by students

Teacher participants mentioned a range of coping strategies that were commonly used by students. There were adaptive coping strategies such as talking to the school counsellors, playing sports, hanging out with friends, talking to parents and friends and so on. The students also frequently used maladaptive coping strategies such as avoidance of problems, overeating, and addiction to novels and computer or mobile games.

*Some students, especially some girls, choose to eat crazily when they were under stress. Really.. some students felt they were venting their unpleasant emotions by eating. (Yue, teacher group, 42 years old, 20 years of teaching experience) …Some outgoing students liked to do sports to release their pressure. (Guo, teacher group, 28 years old, 5 years of teaching experience)* … *for some students with poor grades or the students who did not receive many affirmations, their method was to give up on themselves by indulging in reading novels, playing [computer or mobile] games, and releasing pressure in that way. For example, in the game, they could kill anyone, and vent as much as they liked. (Yue, teacher group, 42 years old, 20 years of teaching experience)*

Teachers thought that the coping strategies, including both adaptive and maladaptive strategies, adopted by students were mostly ineffective. Because the teachers thought the only effective method to reduce stress was closing the gap between their expectations, which could be achieved by either decreasing their expectations relating to study and interpersonal relationships or by increasing their study strategies and interpersonal skills.

*My understanding is that stress is actually the gap between expectations and reality, including academic expectations and interpersonal expectations. For example, in terms of interpersonal relationships, students want all their classmates to recognize them, but they did not have that ability. In order to solve the stress, no matter what coping strategies they used, like singing, dancing and eating, these strategies do not bridge the gap between ideal and reality. What they really need to do is to change themselves or lower their expectations. (Sheng, teacher group, 37 years old, 14 years of teaching experience)*

### Teacher theme 4: recommendations for stress management programs

Teacher participants agreed that, in order to manage stress among Chinese high school students successfully, stress management programs were needed. They outlined the contents and features of such programs. First, the programs should train students with stress coping skills, such as emotion regulation skills and interpersonal skills. Second, career development guidance should be provided to guide students explore their potential future careers and thus make a good choice about universities and majors after graduating from high school. Third, an understanding and support environment in programs. Fourth, parents should be involved.

*On the one hand, in dealing with interpersonal relationships, the program should help students to achieve a certain interpersonal goal and provide students with skills in dealing with interpersonal relationships, enabling them to better deal with conflicts in interpersonal relationships. On the other hand, it would be good to help students learn how to plan their careers. (Zhang, teacher group, 38 years old, 13 years of teaching experience) … But some of the stress is passive, and I think it’s more of a need for an understanding. Whether it is from the parents, or the teachers, no matter how much stressed they feel, as long as there is one person or a group who can think from the students’ perspective, they will feel very happy. (Lin, teacher group, 49 years old, 29 years of teaching experience)*

## Discussions

This is the first study to qualitatively examine the stress experience, stressors, coping strategies and needs of Chinese high school students from the perspectives of both students and teachers. The results of our study suggested that Chinese high school students experienced stress which came from high expectations for academic performance, interpersonal relationships, and family issues. The amount of stress experienced by students had negative impacts on students’ study, emotions, relaxation, and mental health wellbeing. The diverse coping strategies used by students were effective in relieving from stress only in the short term, therefore, more stress coping trainings are needed to assist students manage stress effectively in the long run.

### Stress and stressors

In China, high school education is essential for preparation for College Entrance Examination (CEE) in which the score determines the prestige of university the students can enter. The more prestigious university the students graduate from, the higher salary and better hiring opportunities the students will obtain in labour market [28]. In accordance with previous quantitative findings [16], our study showed that both teachers and students agreed that academic performance is the primary stressor experienced by Chinese high school students. The academic stressors are manifested as highly competitive school environments which put top priority on successes in academic life, including getting good scores and ranking high in exams. Our findings are distinguished from several studies that were conducted in other countries [8, 10, 11, 13], in which family issues and peer relationships were experienced as primary stressors. Previously a study argued that Asian students are under more academic stress because of cultural difference (i.e., the Confucius Heritage Culture in China, Singapore, Japan and Korea) [29]. Our unique study shed light on this difference and pointed out that expectations rather than difficult materials, which is the source of academic stress in Westernized countries, contributed most to academic stress of Chinese high school students.

Both teachers and students agreed that interpersonal stress was another type of stress experienced by Chinese high school students, which was primarily manifested as supressing feelings (e.g., anger in conflicts) in interpersonal relationships. However, only teachers mentioned supressing feelings in romantic relationships as a source of interpersonal stress. The reason might be that adolescents were reluctant to share their romantic relationships/feelings among strangers in focus group interviews. The suppression of feelings revealed in our study distinguished from previous studies, which showed that adolescents from westernized countries experienced interpersonal stress in the form of bullying, teasing and intimidation. This might be explained by cultural difference. Chinese culture value the Confucius *harmony (he xie)* concept in interpersonal relationships [30], which means having physical or verbal conflicts with others is violating and people frequently use emotional suppression in interpersonal relationships [31]. This, nevertheless, indicates the lack of communication skills (e.g., assertive skills) among students. Previous studies have shown that better communication skills is significantly associated with higher social self-efficacy [32], which correlates with lower interpersonal stress [33]. Family stress is another type of interpersonal stress suggested by our study, previous quantitative studies showed that Chinese adolescents frequently experienced family stress [34], our study provides further in-depth information relating to adolescents’ perceptions and experiences regarding how family stress generated and should be addressed. Family environment can buffer stress or be a source of stress for adolescents depending on the parenting styles, family relationships (e.g., harmonious or harsh) [15, 35, 36]. For example, several studies [7, 8, 13] found that when parents adopted strict parenting style, had poor communication skills or divorced, adolescents tend to experience family stress. Similarly, our study showed that most Chinese adolescents experienced family stressor, this includes family issues (e.g., discordant family atmosphere and parental divorce). Additionally, our unique finding suggests that Chinese adolescents also had family stress when they experienced lack of care from parents (for boarding students) and inappropriate parenting styles (e.g., spoiling or too strict). In contrast, our study also showed that when parents are understanding and supportive, adolescents felt that their stress was relieved, suggesting the importance of building understanding and supportive environment in Chinese families.

### Impact of stress on students

According to Yerkes-Dodson law, the right amount of stress can increase motivation and performance, however if the amount of stress exceeds the ability of the individual to cope, it can cause decreased performance and mental health issues [37]. Previous research has shown that stress demotivate high school students in their studies [38], decrease individual’s performance [39] and correlate with self-harm among adolescents [40]. Our study confirms these findings, both teachers and students believed that the stress experienced by students caused study and mental health issues for students. The triangulation of findings from teachers and students revealed the mechanism where students’ cognitive process (i.e., constantly thinking about stressful events during bed time and classes, maladaptive thinking styles) played a critical role. This implies that providing students with strategies to stop rumination and cognitive restructuring is needed in stress coping programmes. In addition, few qualitative research found that some students were aware that stress could motivate them [10, 13]. However, our study did not show this trend. A possible explanation for this might be that the amount of stress experienced by Chinese high school students was excessive.

### Coping strategies

Previous studies have shown that stress coping resources have a buffering effect on the impact of stress on mental health [6]. Previous research has distinguished two kinds of coping strategies: active coping versus avoidant coping, where active coping was defined as improving the situation and thinking of the situation from a different perspective, whilst avoidant coping was defined as avoiding the situation. Previous studies have shown that avoidant coping was significantly associated with increased risk for internalising and externalising problems whilst active coping was associated with reduced risk for these problems [41]. Our findings suggest that regardless of diverse coping strategies that have been used by Chinese adolescents, the primary coping strategies belong to avoidant coping (e.g., engaging in leisure activities), and both students and teachers agreed that these coping strategies were helpful in the short term but not in the long run. The triangulation of results from teachers and students revealed that, firstly, only teachers stated talking to counsellor was an adaptive coping strategy used by students, however, students did not mention that. This might be due to the stigma associated with using mental health services and students were reluctant to admit using mental health services in focus groups. Secondly, teachers thought talking to parents and teachers was an adaptive coping strategy used by students. However, students regarded it as ineffective and sometimes harmful as teachers/parents would not understand them. This difference might be due to the Chinese classroom/family culture where teachers/parents are regarded as authority whereas students are expected to be obedient [42]. This difference also reveals that more equal and effective communications between teachers/parents and students are needed. Previous studies also suggested that adolescents would also use substance (e.g., alcohol, cigarettes, marijuana) as a coping strategy [8, 9]. The deficiencies in effective coping strategies highlights the importance of providing adolescents with effective coping strategies.

### Recommendations for programs

A meta-analysis conducted in 2020 [43] found that school-based programs targeting universal samples were not effective in reducing stress. However, it is important to provide preventive programs for those with emerging problems at schools. Our study revealed important insights into the perspectives of students and teachers regarding their needs and expectations from those preventive programs. According to our results, first, it is important to provide coping skills training including emotion regulation, problem solving and interpersonal skills. These skills have been proven by previous research as vital to reducing stress and improving mental health wellbeing [44–46]. Second, it is crucial that adults (e.g., teachers, parents, and counsellors) think from students’ perspectives without judging them while providing social support for them, otherwise, such support might not be effective and can prevent students from seeking further help. Third, according to the bioecological model proposed by Bronfenbrenner and Ceci [47], children’s development is dynamically influenced by their families, school settings and subculture. Therefore, in order to provide a program that causes dynamic changes in students’ stress experience and coping behaviours, it is important to build a school environment and family culture that values the mental-health well-being of children the same as their academic performance. This requires the involvement of stakeholders in such programs.

### Strengths, limitations, and future research

The strengths of our study include that we recruited both head teachers and students and there was a high degree of confluence in participant responses. Head teachers have many years of teaching experience and have wide contact with a large number of high school students and their families. Involving them enables us to confirm themes identified in the modest number of student focus groups. In addition, involving stakeholders in the development of interventions has been suggested by previous research as valuable process [48]. Another strength is that our sampling was largely based on chance (i.e. randomization), which allows us to recruit most representative participants. Several study limitations should be noted. First, we did not recruit grade 12 students due to administrative restrictions, the reason provided was that grade 12 students were preparing for the college entrance examination, and which made their involvement impractical. However, this population is also the subgroup of high school students under the most stress. Therefore, apply the results of this study to grade-12 students should be done with caution. Future research is warranted to investigate this group of students’ stress experience and needs for support. Second, as head teachers in our participating school were all male, we lack perspectives from female teachers who might hold different opinions regarding students’ stress, coping strategies and needs for support. We suggest future studies to recruit more balanced gender representatives. Third, our study was conducted in a moderate city in China, the results might not be generalised to other big cities and remote areas. Lastly, as each focus group interview lasted around one hour and some participants might need more time to organise their answers, thus some participants might not have enough opportunity to talk about their experiences. Therefore, more confirmatory studies are needed to have a more comprehensive understanding of Chinese high school students’ stress and needs.

### Practical implications

The results of current study shed light on the experience of stress has impact students’ study, health, and wellbeing. Our study also highlights the fact that students are lack of adaptive and effective coping strategies among Chinese high school students. These findings have implications for stakeholders. Most importantly, education system should place the same priority on students’ mental health wellbeing as on academic performance. Second, as our research showed that students feel more stress from expectations and comparison with other students compared with the schoolwork itself, therefore, it is recommended that, when assessing students’ academic performance, Chinese high schools focus more on the improvement of individual student, rather than comparison with other students via ranking. Third, school educators can set regular courses teaching stress coping skills, which address different type of stressors in students’ lives. As our study showed that students have difference preference for stress coping, such courses should cover various types of coping strategies, for example, somatic skills, cognitive skills, behavioural skills to suit the characteristics of different students. Fourth, school counselling centres should help students identify the symptoms of severe stress and provide individual counselling services for such students. To assist parents of high school students, educators can provide online or offline education sessions or posters, which illustrate sources of stress and effective ways to reduce stress for students. Finally, stakeholders should provide an environment which encourage students to talk about their stress and understand students stress experience from the perspectives of students.

## Conclusions

This study is the first to assess perceptions of Chinese high school students and their teachers of students’ stress experience and coping strategies. Chinese high school students experience excessive stress in their daily lives. Their stress comes from school, family, and peer relationships. The stress experience has negative impact on their study, mental health, and wellbeing. However, they are not adequately equipped with necessary effective coping skills to deal with those stressors. Coping skills trainings are needed for Chinese high school students.

## Electronic supplementary material

Below is the link to the electronic supplementary material.

Supplementary Material 1

## Data Availability

The datasets generated and/or analysed during the current study are available from the corresponding author on reasonable request.
